# Low-Grade Inflammation Is Associated with Susceptibility to Infection in Healthy Men: Results from the Danish Blood Donor Study (DBDS)

**DOI:** 10.1371/journal.pone.0164220

**Published:** 2016-10-04

**Authors:** Kathrine Agergård Kaspersen, Khoa Manh Dinh, Lise Tornvig Erikstrup, Kristoffer Sølvsten Burgdorf, Ole Birger Pedersen, Erik Sørensen, Mikkel Steen Petersen, Henrik Hjalgrim, Klaus Rostgaard, Kaspar Rene Nielsen, Henrik Ullum, Christian Erikstrup

**Affiliations:** 1 Department of Clinical Immunology, Aarhus University Hospital, Aarhus, Denmark; 2 Department of Clinical Immunology, Naestved Hospital, Naestved, Denmark; 3 Department of Epidemiology Research, Statens Serum Institut, Copenhagen S, Denmark; 4 Department of Clinical Microbiology, Aarhus University Hospital, Aarhus, Denmark; 5 Department of Clinical Immunology, Copenhagen University Hospital, Rigshospitalet, Copenhagen S, Denmark; 6 Department of Clinical Immunology, Aalborg University Hospital, Aalborg, Denmark; Kinki Daigaku, JAPAN

## Abstract

**Introduction:**

The aim of this study was to examine whether low-grade inflammation (LGI) is associated with a subsequently increased risk of infection.

**Methods:**

We included 15,754 healthy participants from the Danish Blood Donor Study, who completed a questionnaire on health-related items. LGI was defined as a C-reactive protein level between 3 and 10 mg/L. Infections were identified by ICD-10 codes in the Danish National Patient Register and ATC-codes in the Danish Prescription Register. Multivariable Cox proportional hazard analysis was used as the statistical model.

**Results:**

During 53,302 person-years of observation, 571 participants were hospitalized for infection. Similarly, during 26,125 person-years of observation, 7,276 participants filled a prescription of antimicrobials. LGI was associated with increased risk of hospital-based treatment for infection only among men (hazard ratio = 1.60, 95% confidence interval (CI): 1.10–2.34) and specifically infections were abscesses and infections of the skin and subcutaneous tissue. Similarly, LGI was associated with the overall use of antimicrobials among men, and particularly with phenoxymethylpenicillin and broad-spectrum antimicrobials for treatment of urinary tract infections. The difference between men and women was not statistically significant.

**Conclusions:**

In a large cohort of healthy individuals, LGI was associated with an increased risk of infection among healthy male blood donors.

## Introduction

C-reactive protein (CRP) is a well-known acute phase protein and a part of the innate immune response. CRP levels increase rapidly during acute inflammation, and is elevated in several pathological processes, including chronic inflammatory diseases, cancer, and tissue damage [1]. CRP is secreted mainly by hepatocytes under transcriptional control by the cytokine IL-6. CRP recognizes altered self and foreign molecules and activates the complement system, binds to Fc receptors, and acts as an opsonin for various pathogens [2]. CRP could thus be a factor in the clearance of infection.

Accordingly, CRP above 10 mg/L in plasma is routinely used as a marker of infection in the clinical ward. An increased CRP level is associated with several diseases [3], and a CRP level above 10 mg/L is associated with an increased risk of cardiovascular disease [4], rheumatoid arthritis [5], hypertension [6], and colorectal cancer [7].

Slightly elevated CRP levels, also known as low-grade inflammation (LGI), is reported to occur in approximately 10% of healthy individuals [4,8]; a result recently confirmed in the Danish Blood Donor Study (DBDS) [9]. LGI is associated with cardiovascular disease [4] and rheumatoid arthritis [5]. Furthermore, CRP is widely used in cardiovascular risk assessment [4,10] and as a prognostic marker in e.g. non Hodgkin’s lymphoma and diffuse large B-cell lymphoma [11,12]. CRP is, however, also positively associated with several lifestyle factors, such as obesity, smoking, alcohol consumption, and high meat consumption [13]. We recently showed that combined oral contraception (OC) and obesity were strong predictors of LGI in the DBDS study population [9]. We also reported that obesity is associated with a higher risk of infection [14].

LGI may either reflect subclinical infection or it may be a result of immune activation and exhaustion of immune cell subsets with consequently increased infection risk. An association between LGI and risk of subsequent infection has been established in a few studies previously [15–17]. The largest study showed an association between LGI and particularly pneumonia, sepsis, and gram-negative infections [15]. The findings point to CRP as a potential modulator of either risk or severity of infection.

To examine whether LGI is associated with an increased risk of infection, it is necessary to minimize the effect of potential confounders. In this respect, blood donors—who represent a particularly healthy subset of the population—are especially suitable as a study cohort.

## Materials and Methods

### Study population

The Danish Blood Donor Study has been described in detail previously [9,14,18–21].

Briefly, DBDS was initiated in March 2010 as a multicenter public-health study and bio bank (www.dbds.dk). Currently, more than 100,000 blood donors aged 18–67 years participate in the study. Participants filled out a four-page questionnaire on health-related items, regarding current smoking status, alcohol consumption, physical activity, diet, anthropometric measurements, and (among women) use of contraception, childbirth, and menopausal status. Plasma samples were stored and donors granted permission to link their data to public registers.

From 1 March to 31 December 2010, 25,941 participants were included in DBDS. CRP was measured in plasma samples from 18,147 participants included between March and December of whom 15,222 participants were included between March and August and 2,925 participants between September and December.

CRP was measured by a commercially available, high-sensitivity assay on an automated system (Ortho Vitros 5600, Ortho Clinical Diagnostics, Rochester, NY, USA).

Participants were excluded if they had missing responses to any of the following questionnaire items: current smoking status (1,998 participants), height (1,894), and weight (1,800). Furthermore, participants with CRP above 10 mg/L were excluded (141) to prevent possible cases of prevalent infection. In total, 1,159 women and 1,234 men were thus excluded leaving a total of 15,754 participants with corresponding measurements of CRP for the analyses.

For more detailed information on the participants and the measurement of CRP, see Sørensen et al. [9].

### Identification of hospital visits due to infection

To identify the first infection-related hospital visit (including both hospital admission and outpatient care), participants were followed from inclusion via the Danish National Patient Register. The register was established in 1977 [22,23] and holds records of all hospital contacts in Denmark with information on dates of admission and discharge.

Discharge information was based on the International Classification of Diseases, 8th Revision (ICD-8), until the end of 1993, and on the 10th revision thereafter [24].

Date and ICD-10 codes for a discharges with a diagnosis of infection were identified and included apart from chronic infections (other than chronic tonsillitis), condyloma, fungal infections, and parasitic infections.

Diagnosis codes are divided into different types, and we used A (primary diagnosis), B (secondary diagnosis), and G (underlying medical condition).

Infections were grouped into subgroups (previously defined in [14]): abscesses, infections of the skin and subcutaneous tissue, ear- and respiratory tract infections, gastrointestinal infections, urinary tract and pelvic inflammatory diseases only among women.

For specific ICD-10 codes—see S1 File.

### Identification of prescriptions of antimicrobials

Information on antimicrobial prescriptions was collected from The Danish National Prescription Register (DNPR). DNPR was established in 1994 and maintains information on all prescribed drugs dispensed from Danish pharmacies. This information includes the unique personal identification number of the patient, the type of drug according to the Anatomical Therapeutic Chemical classification system, and the date of prescription [25].

Dispensed prescriptions for all antimicrobial agents prescribed for oral treatment of bacterial (J01x) or viral infections (J05x) were identified. Furthermore, national Danish guidelines for primary care were followed for specific calculations on prescriptions resulting from infections of specific anatomical locations [26,27] and previously used in [14]. As a proxy for respiratory tract infections, we evaluated the number of dispensed prescriptions for oral treatment with phenoxymethylpenicillin: J01CE02 and specific macrolides (erythromycin: J01FA01, roxithromycin: J01FA06, and clarithromycin: J01FA09). Phenoxymethylpenicillin is the most frequently used drug against upper respiratory tract infections, whereas macrolides are used in case of penicillin allergy or against mycoplasma pneumonia. To assess skin infections, we used dispensed prescriptions of dicloxacillin: J01CF01 and flucloxacillin: J01CF05. To assess urinary tract infections, we used dispensed prescriptions of pivmecillinam (J01CA08) and sulfamethizole (J01EB02), which are the first-line empiric antimicrobials for treatment of acute, uncomplicated urinary tract infections. In an additional analysis, we included other antimicrobials that can also be used for treatment of urinary tract infections (pivampicillin: J01CA02, ciprofloxacin: J01MA02, nitrofurantoin: J01XE01, and trimethoprim: J01EA01). Ciprofloxacin can also be used for gastrointestinal infections.

Furthermore, an analysis was performed with commonly prescribed broad-spectrum penicillins: amoxicillin (J01CA04) and amoxicillin with enzyme inhibitor (J01CR02). These antimicrobials do not reflect a specific anatomical site.

### Comorbidity

Some major chronic diseases could be potential confounders associated with infection. To assess the burden of disease, Charlson’s Comorbidity Score (CCS) [28] was included in the analyses. This score is a weighted score of 17 major diseases identified by ICD-10 codes in the National Patient Register. CCS includes congestive heart failure, chronic pulmonary disease, cerebrovascular disease, dementia, rheumatic disease, diabetes, renal disease, cancer, liver disease, AIDS, and other diseases. We extended the score by adding hypertension (ICD-10: I10, I111, I13, and I15), gastro-esophageal reflux (ICD-10: K21) [29], and atrial fibrillation (ICD-10: I48) [30], each weighted by one point. The Charlson Index for each participant was calculated as a cumulative score for the 15 years before inclusion. As described previously, we categorized the participants as Charlson Index 0 (no comorbidity), Charlson Index 1 (participants with a score of 1), and Charlson Index 2 (participants with a score above one) [31].

### Definition of low-grade inflammation

We followed common practice by defining LGI as a CRP level above 3 mg/L and below or equal to 10 mg/L [19,32,33].

### Statistical analysis

Multivariable Cox proportional hazards analysis with age as the underlying timescale was used to examine the association between LGI and treatment for infection at a hospital. Study participants were followed from the date of inclusion in DBDS until either the first infection-related contact to the hospital, emigration, death, or end of follow-up (31 December 2013), whichever came first. The same method was used to determine the association between LGI and receiving prescriptions of antimicrobials, however, with follow-up until 31 December 2012.

The results were presented in two models. Model 1 represents a crude Cox proportional hazard analysis without any adjustments. Model 2 includes adjustments for BMI ≥ 30 (yes/no), comorbidity (using CCS defined as above), and current smoking status at inclusion (yes/no). We decided to stratify for sex a priori because of the focus on sex-specific differences discovered in earlier publications by others and us [14,29,34].

Interaction tests between LGI and the predictors were included in all models and removed when confirmed insignificant. Furthermore, proportionality was checked by plotting the observed and the fitted survival curves and log–log plots.

Data are presented as numbers, medians with ranges, or frequency statistics. Risk estimates are presented as hazard ratios (HRs) with 95% confidence intervals (CIs).

Incidence rates are shown as events per year at risk.

Stata/MP 13.1 for windows (StataCorpLP, Texas, USA) was used for statistical analysis.

### Ethics

Oral and written informed consent was obtained from all participants. The Ethical Committee of Central Denmark (M-20090237) approved the study. Additionally, the bio-bank and research database were approved by the Danish Data Protection Agency (2007-58-0015).

## Results

The characteristics of the participants at the time of inclusion have been reported previously [9,14,18–21,35]. Characteristics relevant for this study are presented in Table 1.

**Table 1:** Characteristics of the cohort.

	Women	Men
**Numbers of participants**	7,457 (47.3%)	8,297 (52.7%)
Age≤30	2,367	1,997
30>age≤40	1,736	2,152
40>age≤50	1,801	2,097
50>age≤60	1,152	1,426
Age>60	401	625
**Age, years**	37.8 (27.3; 47.9)	40.0 (30.3; 49.9)
**Weight, kg**	67.0 (61.0; 75.0)	84.0 (76.0; 92.0)
**Height, cm**	169 (165; 173)	182 (178; 187)
**Waist, cm**	82.0 (76.0; 90.0)	92 (86.0; 99.0)
**BMI, kg/m**^**2**^	23.5 (21.5; 26.2)	25.1 (23.3; 27.4)
**Current smoker**	1,281 (17.0%)	1,333 (16.1%)
**Combined oral contraceptive pill (yes)**	2,204 (29.6%)	-
**BMI, categorized, kg/m**^**2**^		
BMI<18.5 (underweight)	79	23
18.5≤BMI<25 (normal weight)	4,822	4,038
25≤BMI<30 (overweight)	1,842	3,386
BMI≥30 (obese)	714	850
**CRP, mg/L**	0.66 (0.17; 1.87)	0.41 (0.05; 1.09)
CRP>3	1,068	494
CRP>5	449	181
**Treated for infection at a hospital**	249	322
**Charlson's score ≥ 1**^**a**^	197	207
**Filled at least one prescriptions of antimicrobials**	4,069	3,207
CRP≤3	0.5 (0.1; 1.2)	0.4 (0.1; 0.9)
3<CRP≤10 (LGI)	0.7 (0.2; 1.9)	0.4 (0.1; 1.1)
CRP>10	12.1 (11.0; 13.3)	11.5 (10.5; 12.6)

Numbers with percentages or medians with interquartile ranges.

^a^ Charlson's index were calculated 15 years prior to inclusion.

There were no differences between the excluded participants and the participants in the study with regard to sex distribution (P = 0.18). For age, however, there was a difference (P = 0.009). Yet, comparing the mean value for age between the two groups; age was 39.7 for the participants in the study and 40.5 for the excluded.

For participants with missing values for smoking, there was no difference with regard to BMI (p = 0.30) or sex (p = 0.06), when compared to participants with available values. However, the participants with missing values were slightly older (40.5 vs. 39.6 years, p = 0.003). For participants with missing values for BMI, there were no differences in distribution of smoking (p = 0.12) or sex (p = 0.18). Again, for age the participants with missing values were slightly older (40.5 vs 39.7 years). We consider the difference in age too small to affect results.

### Low-grade inflammation and hospital-related treatment for infection

The results of the Cox regression analysis are presented in Table 2. During 53,302 years at risk, 571 participants received treatment for infection at a hospital. Overall, LGI was associated with risk of subsequent infection (crude HR = 1.31, CI: 1.03–1.68). However, when additional adjustment for current smoking status and obesity was applied the association waned (adjusted HR = 1.21, CI: 0.94–1.56).

After stratifying for sex, baseline LGI was associated with an increased risk of subsequent infection among men (HR = 1.74, CI: 1.21–2.51) but only a slight tendency was observed among women (HR = 1.18, CI: 0.81–1.59). There was however, not a significant difference between men and women. The difference between men and women was not statistically significant (interaction between sex and LGI: P = 0.116).

**Table 2:** The association between low-grade inflammation and infection.

			WOMEN		MEN			
			^c^ Model 1	^d^ Model 2			Model 1	Model 2
Site of infection	^a^ N	^b^ IR	HR (95% CI)	HR (95% CI)	N	IR	HR (95% CI)	HR (95% CI)
**Infections overall**	249				322			
CRP≤3 (reference)	208	9.6	1	1	290	11.0	1	1
3<CRP≤10 (LGI)	41	11.3	1.14 (0.81–1.59)	1.06 (0.75–1.50)	32	19.3	1.74 (1.21–2.51)	1.60 (1.10–2.34)
**Abscesses**	49				67			
CRP≤3 (reference)	42	1.9	1	1	58	2.2	1	1
3<CRP≤10 (LGI)	7	1.9	0.96 (0.43–2.14)	0.80 (0.35–1.82)	9	5.3	2.66 (1.31–5.39)	2.19 (1.06–4.54)
**Infections of the skin and subcutaneous tissue**	35				90			
CRP≤3 (reference)	31	1.4	1	1	78	2.9	1	1
3<CRP≤10 (LGI)	4	1.1	0.71 (0.25–2.05)	0.87 (0.31–2.49)	12	7.1	2.52 (1.36–4.65)	2.07 (1.09–3.90)
**Ear- and respiratory tract infections**	82				81			
CRP≤3 (reference)	70	3.2	1	1	72	2.7	1	1
3<CRP≤10 (LGI)	12	3.3	0.97 (0.52–1.79)	0.98 (0.52–1.83)	9	5.3	1.90 (0.94–3.81)	1.80 (0.88–3.68)
**Gastrointestinal infections**	50				57			
CRP≤3 (reference)	40	1.3	1	1	51	1.4	1	1
3<CRP≤10 (LGI)	10	1.5	1.53 (0.76–3.07)	1.45 (0.70–2.98)	6	1.6	1.92 (0.82–4.50)	1.81 (0.76–4.33)
**Urinary tract infections and pelvic inflammatory diseases (women only)**	42							
CRP≤3 (reference)	31	1.4	1	1				
3<CRP≤10 (LGI)	11	3.0	1.89 (0.94–3.77)	1.66 (0.81–3.40)				

Multivariable cox proportional hazards analysis was performed with LGI as predictor with age as the underlying timescale.

LGI is defined as a c-reactive protein (CRP) level between 3 and 10 mg/L.

^a^ N: number of cases.

^b^ IR: incidence rate per 1000 person-years.

^c^ Model 1: multivariable cox proportional hazards analysis was performed with low-grade inflammation (LGI) as predictor.

^d^ Model 2: multivariable-adjusted model based on model 1 with additional adjustments for obesity, current smoking status, and comorbidity.

Among men, LGI was most strongly associated with abscesses (HR = 2.66, CI: 1.31–5.39) and infections of the skin and subcutaneous tissue (HR = 2.52, CI: 1.36–4.65). When additional adjustment for current smoking status and obesity was applied, the associations were slightly attenuated for infections overall (HR = 1.60, CI: 1.10–2.34) and similarly for abscesses (HR = 2.19, CI: 1.06–4.54) and infections of the skin and subcutaneous tissue (HR = 2.07, CI: 1.09–3.90).

Additional adjustment for OC use among women did not alter results (data not shown).

### Low-grade inflammation and prescriptions of antimicrobials

The results are presented in Table 3. During 26,125 years at risk, 7,276 participants dispensed at least one prescription of antimicrobials. Overall, LGI was associated with risk of prescriptions (crude HR = 1.27, CI: 1.18–1.37 and adjusted HR = 1.23, CI: 1.15–1.22).

**Table 3:** The association between low-grade inflammation and prescriptions of antimicrobials.

			WOMEN				MEN	
Site of infection			Model 1^c^	Model 2^d^			Model 1	Model 2
Type of prescription	N^a^	IR^b^	HR (95% CI)	HR (95% CI)	N	IR	HR (95% CI)	HR (95% CI)
**Antimicrobials overall**	4,069				3,207			
CRP≤3 (reference)	3,455	332.0	1	1	2,986	200.1	1	1
3<CRP≤10 (LGI)	614	370.1	1.08 (0.99–1.18)	1.05 (0.96–1.15)	221	248.2	1.22 (1.07–1.40)	1.17 (1.02–1.35)
**Phenoxymethylpenicillin**	2,097				1,951			
CRP≤3 (reference)	1,770	134.0	1	1	1,806	108.0	1	1
3<CRP≤10 (LGI)	327	151.0	1.13 (1.00–1.27)	1.06 (0.93–1.19)	145	141.9	1.29 (1.09–1.52)	1.22 (1.03–1.45)
**Erythromycin, roxithromycin, and clarithromycin**	677				559			
CRP≤3 (reference)	550	36.8	1	1	516	27.9	1	1
3<CRP≤10 (LGI)	127	51.8	1.46 (1.20–1.77)	1.31 (1.07–1.60)	43	37.2	1.28 (0.93–1.74)	1.25 (0.91–1.72)
**Dicloxacillin/flucloxacillin**	367				432			
CRP≤3 (reference)	321	20.9	1	1	394	21.0	1	1
3<CRP≤10 (LGI)	46	17.9	0.88 (0.64–1.20)	0.81 (0.59–1.12)	38	32.4	1.54 (1.10–2.16)	1.29 (0.92–1.82)
**Pivmecillinam and sulfamethizole**	1,156				119			
CRP≤3 (reference)	980	68.3	1	1	111	5.8	1	1
3<CRP≤10 (LGI)	176	73.5	1.00 (0.85–1.17)	1.02 (0.87–1.21)	8	6.6	0.97 (0.47–1.99)	1.02 (0.49–2.13)
**Pivampicillin, ciprofloxacin, nitrofurantoin, and trimethoprim**	467				305			
CRP≤3 (reference)	377	24.8	1	1	277	14.7	1	1
3<CRP≤10 (LGI)	90	35.7	1.35 (1.07–1.71)	1.34 (1.05–1.70)	28	23.7	1.61 (1.09–2.38)	1.72 (1.15–2.55)
**Amoxicillin and amoxicillin + enzymeinhibitor**	325				286			
CRP≤3 (reference)	272	17.7	1	1	261	13.8	1	1
3<CRP≤10 (LGI)	53	20.6	1.26 (0.94–1.70)	1.19 (0.88–1.62)	25	21.0	1.37 (0.91–2.07)	1.17 (0.77–1.78)

Multivariable cox proportional hazards analysis was performed with LGI as predictor with age as the underlying timescale.

LGI is defined as a c-reactive protein (CRP) level between 3 and 10 mg/L.

^a^ N: number of cases.

^b^ IR: incidence rate per 1000 person-years.

^c^ Model 1: multivariable cox proportional hazards analysis was performed with low-grade inflammation (LGI) as predictor.

^d^ Model 2: multivariable-adjusted model based on model 1 with additional adjustments for obesity, current smoking status, and comorbidity.

After stratifying for sex, LGI was associated with increased risk of prescriptions for oral antimicrobial agents overall among men (HR = 1.22, CI: 1.07–1.40) but again, only a slightly tendency was observed among women (HR = 1.08, CI: 0.99–1.18). Similar to the analysis above, the difference between men and women was not significant although a tendency was found (interaction between sex and LGI: P = 0.213).

The strongest effects among men were seen with phenoxymethylpenicillin (HR = 1.29, CI: 1.09–1.52), dicloxacillin/flucloxacillin (HR = 1.54, CI: 1.10–2.16), and with pivampicillin/ciprofloxacin/nitrofurantoin/trimethoprim (HR = 1.61, CI: 1.09–2.16). After additional adjustments for current smoking, obesity, and comorbidity, the associations were only slightly reduced: antimicrobials overall (HR = 1.17, CI: 1.02–1.35), for phenoxymethylpenicillin (HR = 1.22, CI: 1.03–1.45), and for pivampicillin/ciprofloxacin/nitrofurantoin/trimethoprim the association increased (HR = 1.72, CI: 1.15–2.55), while the association for dicloxacillin/flucloxacillin were now only a tendency. Among women an association was observed for phenoxymethylpenicillin, macrolides, and for pivampicillin/ciprofloxacin/nitrofurantoin/trimethoprim. However, with additional adjustments for current smoking, obesity, and comorbidity, only the association between LGI and macrolides and LGI and pivampicillin/ciprofloxacin/nitrofurantoin/trimethoprim persisted (see Table 3).

Additional adjustment for OC use among women did not alter results (data not shown).

### The association was not caused by an ongoing infection at inclusion

To ensure that the positive association between LGI and infection was not caused by an ongoing infection at inclusion, specific analyses were done with exclusion of hospital admissions/dispensed prescriptions within 30 days after inclusion. The omission did not change results. Thus, for hospital-related treatment for infection overall the HR associated with LGI for model 2 was 1.61 (CI: 1.11–2.35) among men and 1.07 (CI: 0.76–1.52) among women. Similarly, for dispensed prescriptions of antimicrobials overall the HR for model 2 was 1.16 (CI: 1.01–1.34) among men and 1.04 (CI: 0.95–1.14) among women. Similar findings were found for all subgroups and were consistent to the findings in Tables 2 and 3.

## Discussion

LGI was associated with increased risk of subsequent infection among men in this large cohort of healthy individuals. The association was evident both when defining an infectious event as a hospital contact due to infection and as a dispensed prescription of an antimicrobial. With respect to specific associations, the strongest were found for hospital contacts due to abscesses and to infections of the skin and subcutaneous tissue. Similarly, for dispensed prescriptions of phenoxymethylpenicillin; the most frequently used drug against upper respiratory tract infections and pivampicillin/ciprofloxacin/nitrofurantoin/trimethoprim; commonly used to treat urinary tract infections. An association among women was only found for macrolides and pivampicillin/ciprofloxacin/nitrofurantoin/trimethoprim.

To our knowledge, an association between LGI and later susceptibility to infection has not been described in healthy individuals previously.

The association was not driven by admissions/prescriptions in the month succeeding inclusion. Infections which were subclinical at donation and evolved to clinical infections within short time after donation, were thus not likely the explanation.

Obesity is a major determinant of LGI [36,37] and increased levels of CRP can predict the development of type 2 diabetes, metabolic syndrome [38,39], and insulin resistance [40]. Weight loss leads to CRP decrease [41], and patients with type 2 diabetes are at increased risk of infection [42]. We recently found that obesity is associated with increased risk of infection [14]. The infections associated with obesity are similar to the infections associated with LGI in the current study; namely, abscesses and infections of the skin and subcutaneous tissue. However, LGI was independently associated to infection with only a slight effect on HR estimates of obesity adjustment.

Smoking and alcohol consumption are also determinants of LGI [9,13]. Smoking has previously been associated with risk of infection [43]. We adjusted for current smoking status, and the adjustments had only minor effects on our results.

Additional adjustment for alcohol consumption was included in a separate analysis due to the high percentage of missing answers. The adjustment did not alter conclusions (data not shown).

Similarly, the analyses were adjusted for various forms of comorbidities (using CCS); yet only a small percentage had comorbidities and CCS had no significant impact on our results (see internal and external validity).

Among women, the use of OC was a main cause of LGI [9] and there was no consistent association between LGI and infectious events in this group. All analyses on women were repeated with additional adjustments for OC use (data not shown). The use of OC was not associated with infection and in supplementary stratified analyses among women not taking OC, we did not see stronger associations between LGI and infectious events. Thus, there was no indication that OC use with subsequent LGI should mask an underlying association between LGI and infection.

The association does not seem to be explained by relevant confounders even though residual confounding cannot be ruled out. It can therefore be concluded that LGI was independently associated with risk of infection among men. Only a few other studies have assessed this association and none of these included individuals who were selected to be healthy at inclusion. Because blood donors are thoroughly screened for symptoms at every visit to the blood bank and deferred if not asymptomatic, this study supports that LGI and increased risk of infection are not simply caused by an underlying disease. In stead we share the hypothesis, suggested by another study [15], namely that LGI may represent a low-level immune activation that—if it persists—could ultimately lead to exhaustion of immune cells and a reduced ability to resist infections. Alternatively, donors with subclinical infections develop LGI and also have a tendency to more frequent clinical infections.

### Internal and external validity and Limitations

The validity of the study was strengthened by a well characterized and uniformly asymptomatic study population at baseline. The data collection was structured and participation rate was high. Blood donors must comply with strict criteria to be allowed to donate and are permanently excluded from blood donation if diagnosed with chronic diseases such as diabetes, cancer, hypertension, or even statin-treated hypercholesterolemia. In this study, only 3.2% of the participants had a CCS ≥ 1 and adjustment for CCS or excluding participants with a score ≥ 1 did not affect the results. The participants were followed for a maximum of 2.75 years. Consequently, the time at risk of comorbidity was short. The short follow-up increases the likelihood that the exposure variable was constant during the follow-up, and CRP is relatively constant over time [44]. CRP was only assessed once in this study.

End points was defined by two different approaches to define end points; namely, ICD-10 codes, and dispensed prescriptions of antimicrobials

There are limited data on the validity of infection-related ICD-10 codes; however, a large review showed, that the Danish national patient register is a valuable tool for epidemiological research [45].

ATC-codes reflect the medicine that the participants received. As such, the codes serve as a proxy for the occurrence of an infection; yet it is well known that antimicrobials are often prescribed outside of their proper indication. The manner in which general practitioners treat infections can vary. For mild, uncomplicated infections, some general practitioners are probably more inclined to treat with antibiotics than others, some use broad-spectrum antibiotics where narrow-spectrum antibiotics could have been used, and others are inclined towards waiting for the immune system to clear the infection.

It should be noted that although we observed a direct correlation between LGI and infection susceptibility, this could also be explained by a causal model in which the individuals with LGI have an underlying disorder that leads to more frequent hospital-related treatment for infection and use of antimicrobials. Our inclusion of comorbidity as an adjustment does not indicate that an underlying disorder is the reason for the association.

Blood donors are only allowed to donate if they report that they feel perfectly healthy and they are to report use of medication. At every donation, the blood donors are asked again and whether they have consulted a doctor since last donation. Persons with known diabetes are not allowed to donate blood—but because blood sugar levels are not measured in the blood banks, undiagnosed diabetes and impaired fasting glucose among the participants cannot be ruled out. Similarly, for other diseases associated with LGI, e.g. cancer, cardiovascular disease and autoimmune diseases, no screening apart from the questionnaire is performed. Because these diseases may also alter the susceptibility to infection, we can only conclude that our findings apply to self-reported healthy individuals.

We chose a priori to stratify for sex, however, in combined analysis of men and women only a tendency to a difference in the effect of LGI was found between sexes. Moreover, the high number of tests performed could lead to false positive findings and confirmation in other cohorts would be valuable.

## Conclusion

This first ever study on LGI and subsequent infection among self-reported healthy individuals showed that LGI was associated with subsequent susceptibility to infection among men, mainly involving abscesses and other infections of the skin and subcutaneous tissue, the use of phenoxymethylpenicillin, and antimicrobials for treatment of urinary tract infections.

## Supporting Information

S1 File Diagnostic codes used to identify infection. (DOCX)
