# Supplementary material for: Low-Grade Inflammation Is Associated with Susceptibility to Infection in Healthy Men: Results from the Danish Blood Donor Study (DBDS)
Source: PLoS One. 2016 Oct 4;11(10):e0164220. doi: 10.1371/journal.pone.0164220 (PMC5049789; doi:10.1371/journal.pone.0164220)
Supplement: S1 File — (DOCX) [file pone.0164220.s001.docx]

|  | **ICD-10** | **Exceptions** |
| --- | --- | --- |
| **Infections overall** | The below mentioned ICD-10 codes along with A, B, D738C, D738D, D762, E060B, G00, G01, G02, G039, G04, G05, G06, G07, G531, G630, G734, G940, I00, I01, I02, I30, I320, I321, I33, I38, I39, I400, I410, I411, I412, I430, I520, I521, K112, K113, K12, K140, K230, K770, K858E, K930, M00, M01, M03, M461, M462, M463, M464, M465, M490, M491, M492, M493, M600, M630, M632, M650, M651, M680, M710, M711, M726, M730, M731, M86, M900, M901, M902, and N61. | A06, A07, A33, A630, A65, A66, A67, A68, A69, A881, B079D, B18, B35, B36, B37, B38, B39, B4, B5, B5, B6, B7, B80, B81, B82, B83, B85, B86, B87, B88, B89, B90, B91, B92, B94, I300, I521C, K112B, M031A, M036, M863, M864, M865 and M866. |
| **Abscesses** | A541, B43, D733, E060A, E236A, E321, G06, G07, H000A, H050A, H440A, H600, J340A, J36, J383D, J387G, J390, J391, J398A, J851, J852, J853, K113, K122, K130A, K140A, K209A, K353A, K353B, K570, K572, K574, K578, K61, K630, K650, K750, K810A, K858A, L02, L050, L059, M608A, M868A, M869A, N151, N340, N412, N450, N482, N492A, N619A, N619B, N700A, N700B, N710A, N730A, N730B, N732A, N732B, N733A, N735A, N738A, N738C, N751, N764, and N768A. | A541B, B430, B438, B439, K570B, K570C, K572B, K572C, K574A, K650M, K650N, K650O, and K650P. |
| **Infections of the skin and subcutaneous tissue** | A46, L00-L08, L303, L738H, and L88. | L889 and L889C. |
| **Ear- and respiratory tract infections** | H60, H610, H620, H621, H623, H65, H66, H670, H671, H68, H70, H730, H750, H830, and H940. J00-J06, J09-J18, J20-J22, J340, J350, J36, J383C, J383D, J387B, J387F, J387G, J390, J391, J398A, and J85-J86. | H604, H604A, H605, H605B, H608, H608A, H652, H653, H654, H654C, H661, H662, H663, H681, H701, and H708. J340E, J340F, J340G, and J340H. |
| **Gastrointestinal infections** | A0, K35, DK37, K57, K65, and K67. | [A06, A07, K573C, K573D, K574, K574A, K575, K578, K579, K658A, K658C, K658E, K658F, K658G, and K658H.](http://diagnosekoder.dk/search.aspx?q=section:DK65&filter=0) |
| **Urinary tract infections and pelvic inflammatory diseases** | N080, N109A, N109B, N109C, N12, N136, N151, N160, N288D, N288E, N288F, N290, N291, N30, N330, N340, N341, N342A, N342B, N390, N412, DN45, N481, N482, N49, N511 and N512. N70-N77. | N301, N302, N304, N308, N459B, and N481D. N701, N702, N711, N729B, N729E, N731, N734, N736, N738B, N75, N750, N758, N758B, N759, N761A, N761C, N761E, N763A, N765 and N766. |

**Supporting Information**

**Diagnostic codes used to identify infection**
